# Harvard Veterinary Department

**Published:** 1895-08

**Authors:** 


### COMMENCEMENT EXERCISES.

### Harvard Veterinary Department.

The commencement exercises of the Veterinary Department of Harvard University were held at Saunders's Theatre, Cambridge, on June 26, 1895. The degree of M.D.V. was conferred upon the following students by Charles W. Eliot, LL.D., President of the University: George Adams Clark, 11 Clark Street, Somerville, Mass.; William Walter Jakeman, Halifax, N. S.; Abijah W. Draper, Milton, Mass.; Edward Knobel, Dedham, Mass.; Albert Long, 192 Northampton Street, Boston, Mass.; Charles Eaton McNeil, 509 Columbus Avenue, Boston, Mass.; Robert Douglas Martin, Manchester, N. H.; Edward Roswell Newton, 573 Tremont Street, Hartford, Conn.; Mark Newell North, Prospect Hill, Somerville, Mass.; John Philip O'Leary, 2 Broadway, South Boston, Mass.

